# Editorial: Risk-benefit assessment of foods: Advances in public health

**DOI:** 10.3389/fnut.2022.1089870

**Published:** 2022-11-22

**Authors:** Géraldine Boué, Jeljer Hoekstra, Sara Monteiro Pires, Androniki Naska

**Affiliations:** ^1^Oniris, French National Research Institute for Agriculture, Food and Environment (INRAE), SECALIM, Nantes, France; ^2^National Institute for Public Health and the Environment, Bilthoven, Netherlands; ^3^Risk Benefit Research Group, National Food Institute/DTU, Lyngby, Denmark; ^4^Department of Hygiene, Epidemiology and Medical Statistics, School of Medicine, National and Kapodistrian University of Athens (NKUA), Athens, Greece

**Keywords:** RBA, risk assessment, nutrition, food safety, microbiology, toxicology, One Health

## Introduction

Risk-Benefit Assessment (RBA) of Foods is briskly developing to support complex public health decision-making processes related to nutritional, microbiological and toxicological issues. With the intention to tackle public health matters with a comprehensive and integrative approach, methodologies have evolved, and several challenges were identified and must be overcome to facilitate the use of RBA (1–4). The Research Topic on “Advances in Public Health with Risk-Benefit Assessment of Foods” comprises seven articles including original research and reviews, providing recent progress and perspectives in the field. More specifically, it integrates new RBA case studies that make use of diverse health metrics; suggestions on methodological developments to enhance clarity and transparency in the process; insight of integration in a One Health strategy; and novel considerations of how RBA outcomes could be communicated.

## New RBA case studies

Two case studies were carried out to support dietary guidance related to patterns and frequency of food consumption, in specified contexts for a given population (Fang et al.; Vellinga et al.). All foods have a particular nutritional composition and can also contain microbiological or chemical contaminants. Changing the pattern of consumption of a particular food in a population will have a direct impact on the associated potential risks and benefits for consumers in that population. In the cases studies described (Fang et al.; Vellinga et al.), the aim was to study the nutritional and toxicological impacts of different consumption scenarios of rice and seaweed in two different countries.

Fang et al. carried out a RBA in China among male population aged 40–79 years. Different frequencies of rice consumption were compared to assess health effects related to the intake of selenium (prostate cancer), cadmium (chronic kidney disease) and inorganic arsenic (lung, bladder and skin cancer). The impact was quantified in Disability-Adjusted-Life-Years (DALYs), and the authors concluded that moving from the current consumption of 71.5–105.4 g/day to 50 g/day (scenario 1) or 200 g/day (scenario 2) will differentially affect population sub-groups.

In the Netherlands and Portugal (Vellinga et al.), another RBA was performed to study the potential substitution by seaweed-derived products of 10% of regular foods, among which pasta, bacon and rocket lettuce were selected by the authors as appropriate candidates. The study focused on adults (>18 years old) and considered the exposure to nutrients (iodine and sodium) and heavy metals (arsenic, cadmium, lead and mercury). The main outputs considered for comparison of scenarios were nutrient intakes and exposure to contaminants induced through the substitution scenario and the respective established health based guidance values (HBGVs). Authors reported that the substitution under study may lead to an increased iodine intake and arsenic exposure that would require further investigation.

The selection of health-related metrics to compare intake scenarios is an important step in the RBA process. The two case studies included in this Research Topic highlight two possible candidates, DALYs and HBGVs, and illustrate how to perform an RBA using HBGVs based on exposure assessment (Vellinga et al.) or a RBA using DALYs taking into consideration the health impact that changes in exposure may induce (Fang et al.). The latter provides a more replete comparison, but introduces several sources of uncertainties due to the DALY calculation. The choice of an appropriate strategy to perform a RBA should rely on well-articulated scenarios and the selection of metrics and models that would ensure a balance between data attributes and limitations. Furthermore, it should address the problem in such a manner that the decision-maker will be able to reach a well-informed decision.

## RBA methodological developments

The RBA methodology has been under development for around 15 years; methods and data have substantially improved and experience has accumulated during this last decade. This trend is evidenced by two articles published in this Research Topic (Boué et al.; Thomsen et al.). A key step in a RBA is to select the health outcomes to be studied that are directly related to a food component (nutrient, microbiological or toxicological contaminants) or to the food itself. This selection has been highly variable for instance in fish RBA (5).

In a RBA approach based on food components, the paper by Boué et al. developed a strategy for selecting nutrients, microbiological and toxicological contaminants. This involves the development of a “long list” based on exhaustive search, then a ranking established by a harmonized approach between disciplines to agree on a “short list” and finally establish a “final list” considering data availability and hence feasibility. This structured approach improves the transparency and follow-up of RBA studies as soon as new data become available.

The choice to use a component- or food-based approach might influence the health impact quantified as illustrated by Thomsen et al.. This comparative study focused on health effect characterization and investigated the influence of considering the association of a health effect with: a food component; the food *per se* but not based on substitution analyses; and the food based on specified substitution analyses. This choice demonstrated to highly influence the health impact estimated for two cases (replacement of white by brown rice or unprocessed red meat by vegetables).

Both papers on RBA methodology included in this Topic illustrate the benefit of an integrated approach, consisting of identifying exhaustively, potential components associated with food consumption as well as health outcomes linked directly to the food. Particular attention must however be given to ensure that health outcomes are not counted twice, through components and foods, and that selections need to rely on high quality evidence-based associations.

## RBA in a One Health approach

From a broader perspective, RBA invites us to consider complex food issues holistically, considering all potential health impacts as its main objective is to support decision-making process. However, human health cannot be considered as a sole objective in a sustainable world where human, animal and environment are closely interconnected. A One Health approach is thus necessary as illustrated in the two reviews from Chen et al. and Mantovani et al..

The assessment of feed additives by Mantovani et al. reiterates, through three case studies, the importance of the RBA question definition and the selection of appropriate metrics of comparison as key challenges. In this particular area, considering human and animal risks together with benefits is a prerequisite, and the RBA framework needs to evolve to consider both populations of interest as well as the related environmental impact.

The review by Chen et al. focused on risks and benefits of smallholder livestock production on child nutrition in low- and middle- income countries and supports the need to integrate all parameters considered in the decision process. These include the impact of reducing child undernutrition, the increased exposure to pathogenic bacteria and benefits in terms of production, income and women's empowerment.

## Communication in RBA

Finally, communicating the RBA outcome is of importance not only to ensure an efficient implementation of mitigation strategies based on results obtained, but also to design adequate scenarios in the beginning of the process. The study by Boehm et al. builds upon an RBA to evaluate the substitution of beef with edible insects. It analyzed perceptions and attitudes to support the definition of scenarios and suggests a communication strategy depending on the obtained results. The inclusion of social sciences and even the involvement of stakeholders in the assessment process is increasingly necessary, particularly in the One Health perspective.

## Conclusions

RBA has shown a recent dynamic evolution, with now more than 120 case studies already performed and two more published in this Research Topic (Fang et al.; Vellinga et al.). This evolution is not only reflecting an interest but also the intellectual maturity and enhanced capacity to perform RBAs. Methodological challenges however still need to be addressed, notably with regards to the RBA question, the selection of components to be considered and the development and use of dose-response data (Boué et al.; Thomsen et al.).

In addition, in a One Health perspective, humans, animals and the environment have close interrelations and can barely be considered independently (Chen et al.; Mantovani et al.). Thus, increasing interactions between assessors and managers is necessary to design and evaluate fit for purpose mitigation strategies and to promote healthy, safe and sustainable diets, a key priority in public health.

## Author contributions

All authors listed have made a substantial, direct, and intellectual contribution to the work and approved it for publication.

## Conflict of interest

The authors declare that the research was conducted in the absence of any commercial or financial relationships that could be construed as a potential conflict of interest.

## Publisher's note

All claims expressed in this article are solely those of the authors and do not necessarily represent those of their affiliated organizations, or those of the publisher, the editors and the reviewers. Any product that may be evaluated in this article, or claim that may be made by its manufacturer, is not guaranteed or endorsed by the publisher.

## References

[1] Nauta MJ Andersen R Pilegaard K Pires SM Ravn-Haren G Tetens I. Meeting the challenges in the development of risk-benefit assessment of foods. Trends Food Sci Technol. (2018) 76:90–100. 10.1016/j.tifs.2018.04.00426904635

[2] Assunção R Alvito P Brazão R Carmona P Fernandes P Jakobsen LS. Building capacity in risk-benefit assessment of foods: Lessons learned from the RB4EU project. Trends Food Sci Technol. (2019) 91:541–8. 10.1016/j.tifs.2019.07.028

[3] Pires SM Boué G Boobis A Eneroth H Hoekstra J Membré J-M. Risk Benefit Assessment of foods: Key findings from an international workshop. Food Res Int. (2019) 116:859–69. 10.1016/j.foodres.2018.09.021 30717016

[4] Membré JM Farakos SS Nauta M. Risk-benefit analysis in food safety and nutrition. Curr Opin Food Sci. (2021) 39:76–82. 10.1016/j.cofs.2020.12.009

[5] Thomsen ST Assunção R Afonso C Boué G Cardoso C Cubadda F. Human health risk–benefit assessment of fish and other seafood: a scoping review. Crit Rev Food Sci Nutr. (2021) 62:7479–502. 10.1080/10408398.2021.1915240 33951954

